# Long-Read Genome Assemblies Reveal Extraordinary Variation in the Number and Structure of MHC Loci in Birds

**DOI:** 10.1093/gbe/evaa270

**Published:** 2020-12-26

**Authors:** Ke He, Piotr Minias, Peter O Dunn

**Affiliations:** 1 College of Animal Science and Technology, College of Veterinary Medicine, Zhejiang Agriculture and Forestry University, Hangzhou, China; 2 Department of Biodiversity Studies and Bioeducation, Faculty of Biology and Environmental Protection, University of Łodz, Poland; 3 Behavioral and Molecular Ecology Group, Department of Biological Sciences, University of Wisconsin-Milwaukee, WI, USA

**Keywords:** birds, paralogs, gene duplication, immunity, long-read sequencing, MHC

## Abstract

Our knowledge of the Major Histocompatibility Complex (MHC) in birds is limited because it often consists of numerous duplicated genes within individuals that are difficult to assemble with short read sequencing technology. Long-read sequencing provides an opportunity to overcome this limitation because it allows the assembly of long regions with repetitive elements. In this study, we used genomes based on long-read sequencing to predict the number and location of MHC loci in a broad range of bird taxa. From the long-read-based genomes of 34 species, we found that there was extremely large variation in the number of MHC loci between species. Overall, there were greater numbers of both class I and II loci in passerines than nonpasserines. The highest numbers of loci (up to 193 class II loci) were found in manakins (Pipridae), which had previously not been studied at the MHC. Our results provide the first direct evidence from passerine genomes of this high level of duplication. We also found different duplication patterns between species. In some species, both MHC class I and II genes were duplicated together, whereas in most species they were duplicated independently. Our study shows that the analysis of long-read-based genomes can dramatically improve our knowledge of MHC structure, although further improvements in chromosome level assembly are needed to understand the evolutionary mechanisms producing the extraordinary interspecific variation in the architecture of the MHC region.

**Significance**

Studies of the Major Histocompatibility Complex (MHC) have been limited by extreme levels of gene duplication, which make it difficult to assign loci. Long-read sequencing promises to overcome this difficulty, but, to date, there have not been any comparative studies of its ability to reconstruct the MHC in birds, which have one of the most complex and variable MHCs in vertebrates. Here, we examine the MHC in 34 bird species that have genomes based on long-read sequencing. Across these species, we find that genomes based on long-read sequencing can provide accurate estimates of the number of MHC loci and there is more extensive gene duplication than previously estimated (up to 193 loci in manakins compared with a previously reported average of <25 in birds). Thus, genomes based on long-read technology may provide an easier and more rapid means of studying MHC evolution in birds.

## Introduction

Next-generation sequencing (NGS) has enabled the assembly of over 1,345 vertebrate genomes (GenBank accessed September 9, 2019). Despite the large number of genomes, their quality is limited by the relatively short reads (usually <250 bp) used to construct them (typically from Illumina sequencing by synthesis). This leads to difficulties constructing genomes in regions with paralogs and repetitive elements. Third-generation sequencing (TGS) with “long-read” technology has the potential to alleviate these problems. Pacific Biosystems (PacBio) and Oxford Nanopore are both leaders in this field, and their sequencers can generate reads over 10 kb, which makes TGS ideal for assembling genomes in areas with repetitive elements (Merker et al. 2018; van Dijk et al. 2018) and generating long haplotype blocks (Malar et al. 2019). TGS has already been used in some studies of complex gene families with numerous duplicated copies (i.e., paralogs) (Westbrook et al. 2015; Jain et al. 2018), such as the Major Histocompatibility Complex (MHC). For example, in cynomolgus macaques (*Macaca fascicularis*), TGS provided full-length sequences of MHC class I alleles, which previously had to be assembled from shorter NGS reads (Karl et al. 2017). In Alpine chamois (*Rupicapra rupicapra*) short NGS reads alone were not sufficient to assemble the MHC class II DRB locus, but 9 kb of the locus could be assembled with the aid of a scaffold constructed from nanopore long read sequences (Fuselli et al. 2018). These studies suggest that we might be able to study MHC structure from genomes based on TGS alone. However, to date, this has not been tested at a broad scale.

The class I and II genes of the MHC code for proteins that recognize antigens and present them to lymphocytes, initiating an adaptive immune response. The class I and II genes are highly polymorphic and contain many paralogs. The polymorphism of the MHC is thought to be primarily the result of coevolution between species and their pathogens, which are selected to evade detection by the host MHC (Klein 1986). Sexual selection, maternal–fetal interactions, and demographic processes, such as genetic drift can also affect the evolution of the MHC and, hence, the number of loci (Piertney and Oliver 2006). More recently, computer simulations suggest that complex interactions between three factors (pathogen richness, the intrinsic cost of expressing additional MHC variants, and pathogen mutation rate) drives the number of MHC genes in the genome (Bentkowski and Radwan 2019). These interactions can generate variation in the number of MHC loci within species (paralogs) as well as between species (orthologs). Consistent with this hypothesis, the number of MHC loci in birds was correlated with lifespan and migratory behavior, which are presumably related to exposure to pathogens (Minias et al. 2018a).

In addition to extensive variation in the number of gene copies, there is also remarkable variation in the arrangement of loci (gene structure) between and within species, especially in birds (Balakrishnan et al. 2010; Ekblom et al. 2011; Tsuji et al. 2017; Lan et al. 2019). Most of our knowledge of the structure of the avian MHC comes from chicken-like birds, the Galliformes. A “minimal and essential” MHC model was supported in studies of domestic chickens (Kaufman et al. 1999), but in other Galliformes the number and arrangement of MHC loci is quite variable (Hosomichi et al. 2006; Ye et al. 2012; Eimes et al. 2013). MHC gene duplication and pseudogenes have limited our understanding of MHC gene structure to just a handful of species outside the Galliformes (e.g., Edwards et al. 1998;  Hess et al. 2000; Chen et al. 2015; Tsuji et al. 2017; Lan et al. 2019).

One general pattern that has emerged from MHC studies in birds is that most nonpasserine species appear to have only a few loci, whereas passerine species appear to have numerous duplicated loci (Minias et al. 2018a). However, most of these studies can only estimate the minimum number of MHC loci in a species, because PCR primers that amplify single loci are only available for a handful of species, and, as a consequence, the number of loci has been estimated as the number of different sequences amplified with a particular PCR primer set divided by two (i.e., assuming heterozygosity at every locus). Although the minimum number of loci in a species is important, it does not provide accurate information about gene number or arrangement. Even in the zebra finch (*Taeniopygia guttata*), which has one of the most studied bird genomes, both MHC class I and IIB genes have been identified on chromosome 16, but the physical arrangement and number of gene copies is not clear (Ekblom et al. 2011). Thus, the recent availability of genomes based on ultralong reads from TGS now promises the ability to explore the diversity of MHC genes in greater detail and across a wider range of birds (O’Connor et al. 2019).

The primary aim of this study was to examine the number and structure of MHC loci across bird species using recent genomes, particularly those based on TGS. We found MHC loci using BLAST searches of publicly available genomes that we queried with short MHC consensus sequences constructed from the NCBI database. Our first goal was to determine whether we can obtain more accurate estimates of the number of MHC loci in birds from analysis of the newer TGS-based genomes than the older NGS-based genomes, or the PCR-based methods used in many previous studies of populations. Our second goal was to describe the variation in the number and arrangement of MHC loci between species of birds. In particular, we wanted to verify the higher number of MHC loci in songbirds (passerines) than in other species, which was previously suggested from PCR-based studies of populations (Minias et al. 2018a).

## Materials and Methods

### Constructing Consensus MHC Sequences

**Fig. 1:**
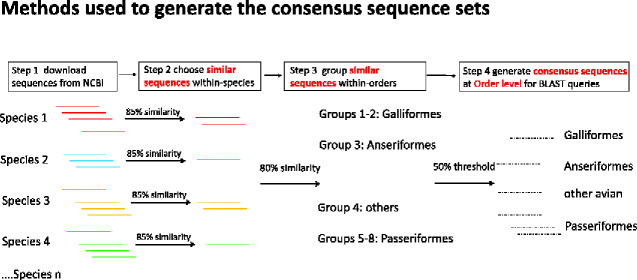
Steps used to generate the avian MHC consensus sequences. All known MHC sequences were downloaded from NCBI (see Materials and Methods). Next, we used Geneious software to align sequences from the same species and then group them into clusters with >85% similarity (Step 2). A random sequence was chosen from each of these species level clusters for further clustering (Step 3). Clustering of the sequences from step 2 (at >80% similarity) produced eight new clusters (“groups”) at Class I. Two of these groups were within the Galliformes, one was within the Anseriformes, three were within the Passeriformes and one contained a mix of species in other Orders. We call this “Order level” clustering since the groups roughly fall within different Orders. At Class II, 14 clusters were formed at Step 3 with five clusters in the Galloanserae, six clusters in the Passeriformes, and three clusters in other avian Orders. At the last step (#4), we used a 50% similarity threshold to group sequences into clusters. Then we used the consensus sequences generated by Geneious as queries in BLAST searches of genomes (see fig. 2). The consensus sequences had some degenerate nucleotides, to allow some variation in sequence matching. The dotted lines represent consensus sequences from Geneious, whereas solid lines indicate original sequences (downloaded from NCBI).

We created consensus sequences for MHC class I and II (fig. 1), so we could use them to search for MHC loci using BLAST searches (Johnson et al. 2008). We started by downloading a total of 1,481 MHC class I and 518 MHC class II sequences of birds from the NCBI database on April 20, 2019 (Step 1 in fig. 1) using “MHC,” “class I,” “class II,” or “mRNA” as search terms (see supplementary section 1 and table S2, Supplementary Material online). Our analysis focused on three exons (2, 3, and 4) in both MHC class I and II that encode the extracellular domains and are involved in peptide binding, particularly exons 2 and 3 of class I and exon 2 of class II. Other exons were excluded because they were short (<100 bp) and generated consensus sequences with a large number of degenerate sites. To produce the final “consensus” sequences at the Order level (fig. 1), we performed sequential alignments of the sequences at three levels, first within species (Step 2 in fig. 1), then within each Order (or Orders, Step 3 in fig. 1), and finally across all Orders (i.e., all species, Step 4 in fig. 1). Sequences were aligned by MUSCLE (Edgar 2004) using default settings in Geneious version 10.0.5 (Kearse et al. 2012) and the threshold levels of nucleotide similarity were set as ≥85%, ≥80%, and 50% for within-species, within-Order and across all Orders, respectively (fig. 1). Details of generating the consensus sequences are in supplementary section 2, Supplementary Material online. At the end of these steps, we had an Order level consensus sequence set, consisting of 8 class I sequences and 12 class II sequences, which were then used as query sequences in BLAST searches (supplementary section 4, Supplementary Material online).

### Estimating the Number of MHC Loci with BLAST Searches

We estimated the number of MHC loci as the number of BLAST hits that contained all three exons (2, 3, and 4) within 2 kb of each other. This maximum distance (2 kb) between exons was chosen based on the distance between exons in published MHC sequences listed in supplementary table S1, Supplementary Material online. Each BLAST search was conducted separately for each exon and class (I or II). We searched for stop codons in exon 3 of class I and exon 2 of class II, which encode the peptide-binding sites, as they may have been functional in the past and then silenced (fully or partially).

### Verifying the Accuracy of Consensus MHC Sequences

**Fig. 2:**
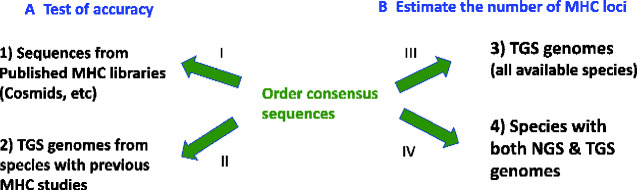
(*A*) Tests of the accuracy of our consensus sequences for BLAST and (*B*) Estimating the number of MHC loci in TGS and NGS genomes. The Order level consensus sequences (see figure 1) were used for four comparisons as indicated by the arrows and Roman numerals. See Materials and Methods for more details.

We used two types of comparisons to test the accuracy of these consensus sequences to estimate the number of MHC loci in each species (part A of fig. 2). First, we compared the number of known MHC loci in published libraries (e.g., cosmids, BACs, fosmids and lambda phage clones) of eight species with the number of loci we estimated from BLAST hits of those same libraries using our Order level consensus sequences (arrow I in fig. 2). Details of each library are in supplementary table S1, Supplementary Material online. Second, we compared the estimated number of MHC loci from published population level (PCR-based) studies to the number of MHC loci we estimated with BLAST searches of TGS-based genomes of the same species (arrow II in fig. 2). We obtained TGS-based genomes by searching the Genome database at NCBI and Vertebrate genomes project (VGP, vertebrategenomesproject.org; accessed on October 23, 2019). Of the 153 species of birds with genomes, just 34 species had genomes based on TGS. Only 13 of these 34 species had MHC class I or class II sequences available from previous PCR-based studies of the MHC (table 1; supplementary table S3, Supplementary Material online has the complete list of genomes and their accession numbers).

**Fig. 3:**
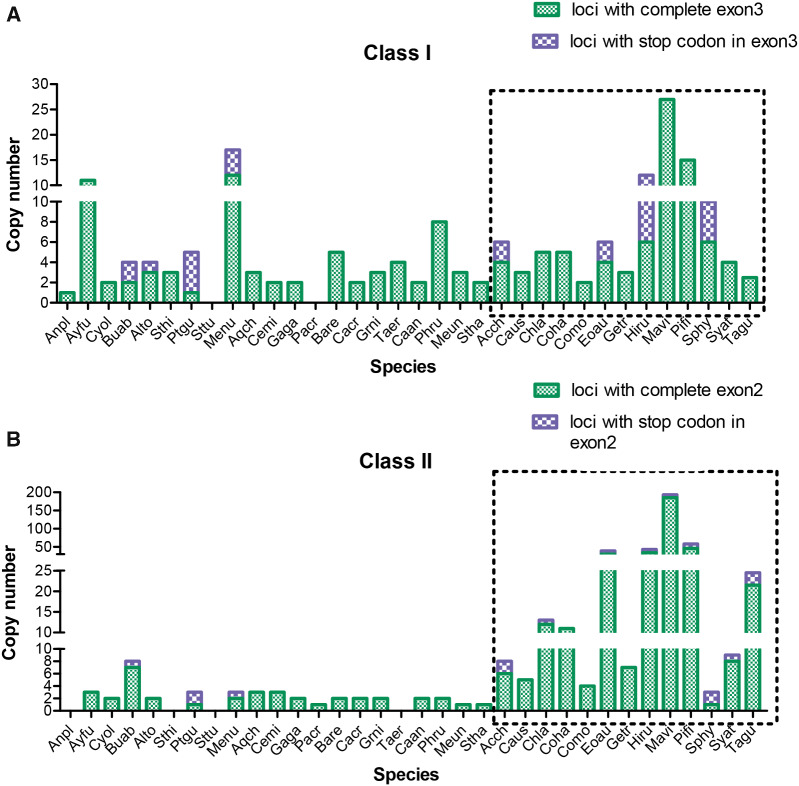
Number of (*A*) class I and (*B*) class II loci in 34 TGS-based genomes. Missing columns indicate no data were available. Means are presented if there were several published values. Passeriformes are indicated by the dotted box. Species names correspond to the first two letters of the Genus and species given in table 1.

**Table 1:** Published and Predicted Number of MHC Class I and Class II loci in TGS-Based Genomes

			Class I		Class II	
Order	Species	Species-Common Name	*N* Published	Order Consensus BLAST	*N* Published	Order Consensus BLAST
Anseriformes	*Anas platyrhynchos*	Mallard	2	1		0
	*Aythya fuligula*	Tufted duck		11		3
	*Cygnus olor*	Mute swan		2		2
Galliformes	*Centrocercus minimus*	Gunnison sage-grouse	1	2	2	3
	*Gallus gallus*	Chicken	2	2	2	2^a^
	*Pavo cristatus*	Indian peafowl		0	3	1
Phoenicopteriformes	*Phoenicopterus ruber*	American flamingo	6	8	2	2
Columbiformes	*Streptopelia turtur*	European turtle dove		0		0
Pterocliformes	*Pterocles gutturalis*	Yellow-throated sandgrouse		5		3
Musophagiformes	*Tauraco erythrolophus*	Red-crested turaco		4		0
Apodiformes	*Calypte anna*	Anna's hummingbird		2		2
Gruiformes	*Balearica regulorum*	Gray crowned crane		5	2	2
	*Cariama cristata*	Red-legged seriema		2		2
	*Grus nigricollis*	Black-necked crane		3	1	2
Charadriiformes	*Alca torda*	Razorbill		4		2
	*Sterna hirundo*	Common tern		3		0
Accipitriformes	*Aquila chrysaetos*	Golden eagle		3	2	3
Bucerotiformes	*Bucorvus abyssinicus*	Abyssinian ground hornbill		4(2)		8(1)
Coraciiformes	*Merops nubicus*	Carmine bee-eater		17(5)		3(1)
Psittaciformes	*Melopsittacus undulatus*	Budgerigar		3		1
	*Strigops habroptila*	Kakapo		2	2	1
Passeriformes	*Acanthisitta chloris*	Rifleman		6		8
	*Catharus ustulatus*	Swainson's thrush		3		5
	*Chiroxiphia lanceolate*	Lance-tailed manakin		5		13(1)
	*Corvus hawaiiensis*	Hawaiian crow		5	9^b^	11
	*Corvus moneduloides*	New Caledonian crow		2	9^b^	4
	*Eopsaltria australis*	Eastern yellow robin		6(2)		39(9)
	*Geothlypis trichas*	Common yellowthroat	7	3	16^c^	7
	*Hirundo rustica*	Barn swallow		12(6)		43(10)
	*Manacus vitellinus*	Golden-collared manakin		27		193(8)
	*Pipra filicauda*	Wire-tailed manakin		15		68(12)
	*Sporophila hypoxantha*	Tawny-bellied seedeater		6(4)		3(2)
	*Sylvia atricapilla*	Eurasian blackcap		4		9(1)
	*Taeniopygia guttata*	Zebra finch	1	2.5^d^	11	24.5(3)

Note.—Numbers outside the parentheses are the total number of loci, including sequences with stop codons (pseudogenes), whereas the numbers inside the parentheses are just the number of loci with stop codons. The number of loci from previously published PCR-based studies is indicated in “*N* published” (see supplementary table S4, Supplementary Material online for references). The number of loci predicted in this study using BLAST searches of TGS-based genomes is indicated in “Order consensus BLAST”.

a For chicken, our estimate (2) excludes four nonclassical loci with lower similarity.

b The number of loci was estimated from published studies of the same genus.

c For common yellowthroat we estimated 16 class II loci based on Whittingham et al. (2015), which used more conservative allele scoring methods and had larger sample sizes than previous studies of this species.

d For zebra finch, the numbers of loci were estimated from the average of several genomes (GCA_000151805.2, GCA_002008985.2, bTaeGut1.pri.cur.20181023.fasta.gz and bTaeGut2.pri.cur.20181019.fasta.gz).

We also conducted the BLAST searches mentioned above with consensus sequences constructed only from the same species (“within species” sequences in Step 2 of fig. 1), rather than the same Order. Our results with these “within species” sequences were similar to those using the Order level sequences, so subsequent analyses are restricted to the Order level consensus sequences (details in supplementary section 3, Supplementary Material online).

### Predicting the Number of MHC Loci in NGS- or TGS-Based Genomes

To predict the number of MHC loci in species with TGS- and NGS-based genomes, we conducted two analyses. First, we used the Order level sequences to search for MHC loci in the TGS-based genomes of all available species (arrow III, fig. 2). This included species that had previous MHC studies (the same set as used in the arrow II comparison in fig. 2), as well as additional species with no previous studies of the MHC. Second, we performed BLAST searches for MHC loci in the genomes of each species that had both NGS- and TGS-based genomes (arrow IV, fig. 2). Eleven species met this requirement (supplementary table S5, Supplementary Material online).

### Analyzing MHC Duplication and Structure in TGS Genomes

We also examined gene duplication and structure in the TGS-based genomes. Here, we focused mostly on passerines, which appear to have a large number of loci and relatively unknown MHC structure. We searched for duplicated genes and regions with a self-dotplot made with EMBOSS 6.5.7 in Geneious. We considered groups of all three exons (2, 3, and 4) to be a locus and focused on multilocus contigs (having two or more MHC loci). To predict the identity of additional genes on these multilocus contigs, we used the online version of the program Maker (http://www.yandell-lab.org/software/mwas.html). In the Maker analyses of passerines we used a protein reference file from the zebra finch (GCF_003957565.1_bTaeGut1_v1.p_protein). For the Maker analysis of nonpasserines, we used a chicken protein reference (GCF_000002315.6_GRCg6a_protein). To visualize the structure of the MHC, we extracted a region around the predicted class I or II loci (10 kb upstream and downstream from the loci) and plotted the gene location using gggenes (https://github.com/wilkox/gggenes), an extension for ggplot2 (Wickham 2016) in R 3.5.2 (R Development Core Team 2018). Only regions with classical MHC class I or II and associated genes were chosen for plotting. All other statistical analyses were conducted with JMP Pro v. 14.3 (SAS Institute 2018). We used nonparametric tests in analyses of highly skewed data.

## Results

### Construction of Consensus Sequences

We generated eight class I consensus sequences (237–821 bp) and 12 class II consensus sequences (157–747 bp) at the Order level from all the avian MHC sequences in GenBank that were not part of a genome assembly (<2 kb; see supplementary section 4, Supplementary Material online). These consensus sequences were then used in all of our subsequent BLAST searches.

### Accuracy of Our Estimates of the Number of MHC Loci

Our first test of accuracy (arrow I, fig. 2) revealed a perfect match between the number of class I and II loci found in 17 published MHC libraries (e.g., cosmids, BACs) and our estimated number using BLAST searches of the same libraries (supplementary table S1, Supplementary Material online). The number of MHC loci varied from one to five in class I and one to seven in class II (supplementary table S1, Supplementary Material online). Sequence similarity ranged from 84% to 95% in class I and 83 to 94% in class II. We were also able to identify previously reported pseudogenes in the sequences of quail (AB078884), Oriental stork (*Ciconia boyciana*; LC180358) and mallard (AY885227; see results in supplementary section 5, Supplementary Material online).

Our second test of accuracy (arrow II, fig. 2) compared our estimates of the number of MHC loci from searches of TGS-based genomes with previous estimates from PCR-based studies of populations of the same species. At MHC class II, our estimates of the number of loci in 12 TGS-based genomes were positively correlated with previously published estimates from the same species (*r *=* *0.64, *n *=* *12, *P *=* *0.026; table 1). The largest discrepancies in the number of loci were at class II in zebra finch (11 published vs. 24.5 estimated here) and common yellowthroat (16 published vs. 7 estimated here). Without these two cases, the correlation was much stronger (*r *=* *0.77, *n *=* *10, *P *=* *0.009). For class I, there were just six species with previously published MHC data, so although the correlations between our estimates and previous estimates of the number of loci were also strong, they were not significant (*r *=* *0.63, *n *=* *6, *P *=* *0.18; table 1).

We found stop codons (potential pseudogenes) in the MHC loci of 29% of species (10/34, table 1). Five species had stop codons in at least five loci (table 1). Nonetheless, our estimate of the number of MHC loci did not change appreciably (median difference = 0) after removing loci with stop codons. In fact, the correlation between our estimates of the number of class II loci from TGS-based genomes and previous estimates from PCR-based studies (our second test of accuracy, arrow II, fig. 2) was slightly higher after removing loci with stop codons (*r *=* *0.66, *n *=* *12, *P *=* *0.019; table 1).

### Number of MHC Loci in Species with TGS-Based Genomes

Next we estimated the number of MHC loci in all species that had a TGS-based genome, including species without a published MHC study (arrow III, fig. 2). This allowed us to add 17 more TGS-based genomes from online sources for a total of 34 species (table 1). The median number of loci per species was 3.5 loci at both class I and II. Accordingly, there was no difference between the number of class I and II loci in a paired analysis within each species (table 1; *n *=* *34 species, Wilcoxon signed rank = 55.5, *P *=* *0.35). After removing loci with stop codons, there was also no difference between the number of class I and II loci within species (table 1; Wilcoxon signed rank = 59, *P *=* *0.32). Across species, there was a positive correlation between the number of class I and II loci (Spearman *rho* = 0.59, *P *<* *0.001), so species with large numbers of class I loci also tended to have large numbers of class II loci, and species with relatively few loci had low numbers at both class I and II. We also examined several characteristics of the 34 TGS-based genomes to see if they were related to our estimates of the number of MHC loci. There was no correlation between our estimate of the number of MHC loci and genome coverage (*r*^2^ < 0.02), scaffold N50 (*r*^2^ < 0.07) or the number of scaffolds (*r*^2^ < 0.07) for either class I or II. This suggests that the quality or contiguity of genomes did not bias our results; however, we made a closer examination of the MHC in manakins because of their extraordinarily high numbers of loci.

Across the 34 genomes (table 1), the most loci were found in golden-collared manakin (*Manacus vitellinus*) with 27 class I loci and 193 class II loci, followed by the wire-tailed manakin (*Pipra filicauda*) with 15 class I and 68 class II loci (fig. 3). In contrast, the lance-tailed manakin (*Chiroxiphia lanceolata*) had numbers of loci that were closer to the averages for all species (5 and 14 loci for class I and II, respectively). One possible explanation for the large number of loci in some manakins is that the MHC region of their genome was not as well assembled as in some other TGS-based genomes, which could produce more contigs with fewer loci per contig. In fact, the golden-collared manakin had the lowest contig N50 of any passerine in our sample (0.29 Mb; average for all species = 10.6 Mb). The wire-tailed manakin also had a relatively low contig N50 (1.6 Mb), whereas the lance-tailed manakin contig N50 (18.5 Mb) was well above the average of all species. As a consequence, the wire-tailed and golden-collared manakins also had a larger number of contigs with MHC BLAST matches than most other species. Despite these differences, manakins had similar numbers of exons encoding the peptide binding regions on each contig (1.3–6.5 exons per contig in manakins vs. 2.5–3 in other species). For more details see supplementary section 7, Supplemental Material online.

### Number of Loci in Passerines and Nonpasserines

Passerines had more loci per species than nonpasserines at both class I (medians: 5 vs. 3, respectively; Mann–Whitney test, *Χ*  ^2^ = 5.2, *P *=* *0.02) and class II (medians: 11 vs. 2, respectively; Mann–Whitney test, *Χ*  ^2^ = 21.5, *P *<* *0.001). Note that in six cases we found partial matches for exons 2–4 with our BLAST searches, but the locations were not close to each other (<2 kb), so we did not count them as complete loci, and, as a consequence, the number of loci appear as zeros in table 1 (for details see supplementary section 8, Supplementary Material online). The difference between passerines and nonpasserines at class II was not due to nonfunctional loci with stop codons, because after excluding them, the number of loci per species was still higher in passerines than nonpasserines (medians: 11 vs. 2, respectively; Mann–Whitney test, *Χ*  ^2^ = 18.1, *P *<* *0.001). At class I, the number of loci became more similar after excluding loci with stop codons (medians: 4 vs. 3, respectively; Mann–Whitney test, *Χ*  ^2^ = 3.6, *P *=* *0.059).

### Comparison of TGS- and NGS-Based Genomes

There were 11 species with both TGS- and NGS-based genomes that we could use to compare numbers of MHC loci (arrow IV, fig. 2). In a direct comparison of these genomes, we found more class I and II loci in TGS-based than NGS-based (short read) genomes, most of which had zero MHC loci (supplementary table S5, Supplementary Material online). As a consequence, there was no correlation between the number of MHC loci found in TGS and NGS-based genomes for class I (Spearman *rho* = –0.21, *n *=* *11, *P *=* *0.53), or class II (*rho* = 0.52, *n *=* *11, *P *=* *0.10). We also compared the number of MHC loci estimated in previous PCR-based studies of populations with our estimates from BLAST searches of both the NGS and TGS-based genomes. Here, the correlation between the number of MHC class II loci we estimated in NGS-based genomes and the number based on PCR-based studies was lower (*r *=* *0.254, *P *>* *0.5; supplementary table S6, Supplementary Material online) than the corresponding correlation using TGS-based genomes (*r *=* *0.64; see above). At class I, the correlation between the number of loci we estimated in NGS-based genomes and the number based on PCR-based studies was negative (*r* = –0.420), but there were only four species available with data, so we did not examine it statistically (see supplementary table S6, Supplementary Material online).

### Arrangement of MHC Loci

**Fig. 4:**
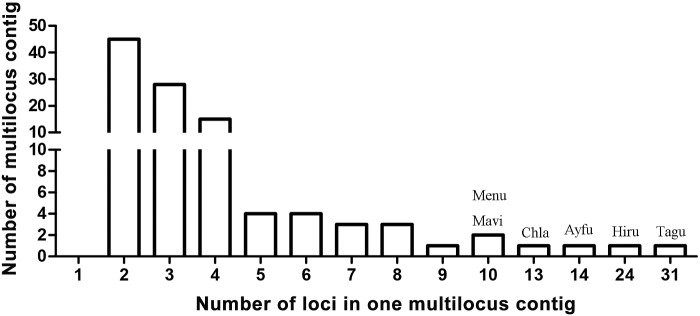
The distribution of multilocus MHC contigs across 34 species. There were 31 loci in *Taeniopygia guttata* cluster 1, 24 loci in *Hirundo rustica* cluster 1, 14 loci in *Aythya fuligula* cluster 1, 13 loci in *Chiroxiphia lanceolata* cluster 1, 10 loci in *Merops nubicus* contig 1, and 10 loci in *Manacus vitellinus* contig 3*.* The number of contigs with a single locus (*N* = 244) was not included for clarity.

To study the arrangement of MHC loci we examined 109 contigs with at least two loci (multilocus). There were 102 multilocus contigs after excluding loci with stop codons (this exclusion did not change the results substantially; for details see supplementary section 9, Supplementary Material online). The multilocus contigs had an average of 3.9 loci per contig (range: 2–31; median = 3 loci; fig. 4, supplementary table S8, Supplementary Material online). Most species had one (*n *=* *13) or two (*n *=* *9) multilocus contigs in their genomes, but the manakins contained many more with 19 in wire-tailed manakin and 27 in golden-collared manakin. Across all species, only 35 of the 109 multilocus contigs had both class I and class II loci, and these were found more often in passerines (median = 2 per species) than nonpasserines (median =1 per species; *Χ*  ^2^ = 4.96, *P *=* *0.03). Overall, there were more multilocus contigs per species in passerines (median = 3) than nonpasserines (median =1; *Χ*  ^2^ = 11.0, *P *<* *0.001), and these were most often contigs with only class II loci, primarily because of the large number in wire-tailed (*n *=* *15 class II contigs) and golden-crowned (*n *=* *21 class II contigs) manakins.

**Fig. 5:**
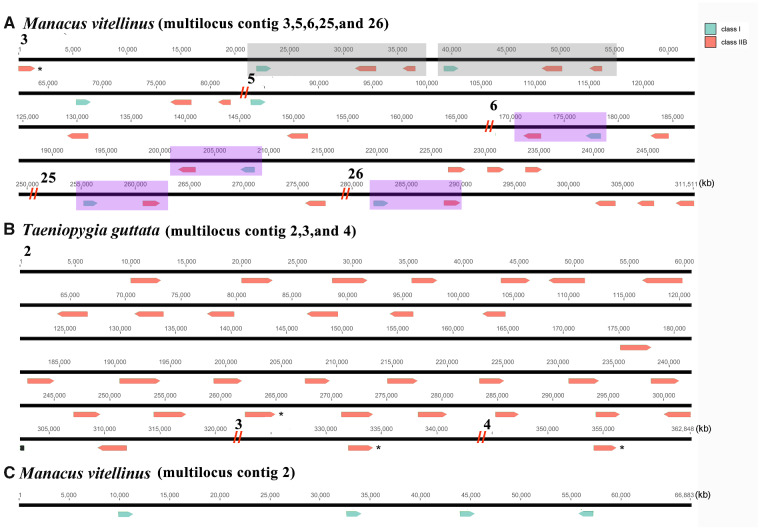
Gene duplication pattern of MHC genes in (*A*) *Manacus vitellinus* (class I and II coduplicated event), (*B*) *Taeniopygia guttata* (class II duplication event), and (*C*) *Manacus vitellinus* (class I duplication event). Contigs were concatenated to display gene locations, and double slashes indicate breaks between different contigs. The genes with stop codons (pseudogenes) are marked with *. Duplicated units are in gray and purple. The figures only show the novel results, rather than all contigs.

The largest number of loci in a single contig was 31 class II loci in the zebra finch (figs. 4 and 5 and supplementary table S8, Supplementary Material online). Here, we focused on the latest TGS-based genome of the zebra finch (bTaeGut1.pri.cur.20181023), which had high numbers of both class I and II loci, and most of them were in one contig. The species with the next largest numbers of loci in a single contig were barn swallow (24 loci), tufted duck (*Aythya fuligula*, 14), lance-tailed manakin (13), carmine bee-eater (10), and golden-collared manakin (10).

There were two distinct patterns of MHC structure in the 13 species of passerines with long-read TGS genomes when we examined their self-dotplots (fig. 5 and supplementary fig. S1, Supplementary Material online). First, most species had duplication of separate class I or class II contigs, but not both class I and II together. The one exception was in the golden-collared manakin where there were duplicated units composed of both class I and class II loci. These duplicated units can be seen in the shaded blocks in figure 5*A* and the parallel lines in the self-dotplots (supplementary fig. S1*a*, Supplementary Material online). Although there were contigs containing both class I and class II loci in other species, they were not duplicated together. The second pattern consisted of numerous copies of class I and class II loci in a single multilocus contig, as shown by contig 2 in the zebra finch and contig 2 in golden-collared manakin (fig. 5*B, C* and supplementary fig. S1*b*, Supplementary Material online). Multiple copies of class II loci were also detected in multilocus contig 1 (19 copies of class II) and contig 5 (6 copies of class II) of barn swallow, and multilocus contig 13 (5 copies of class II) of wire-tailed manakin.

**Fig. 6:**
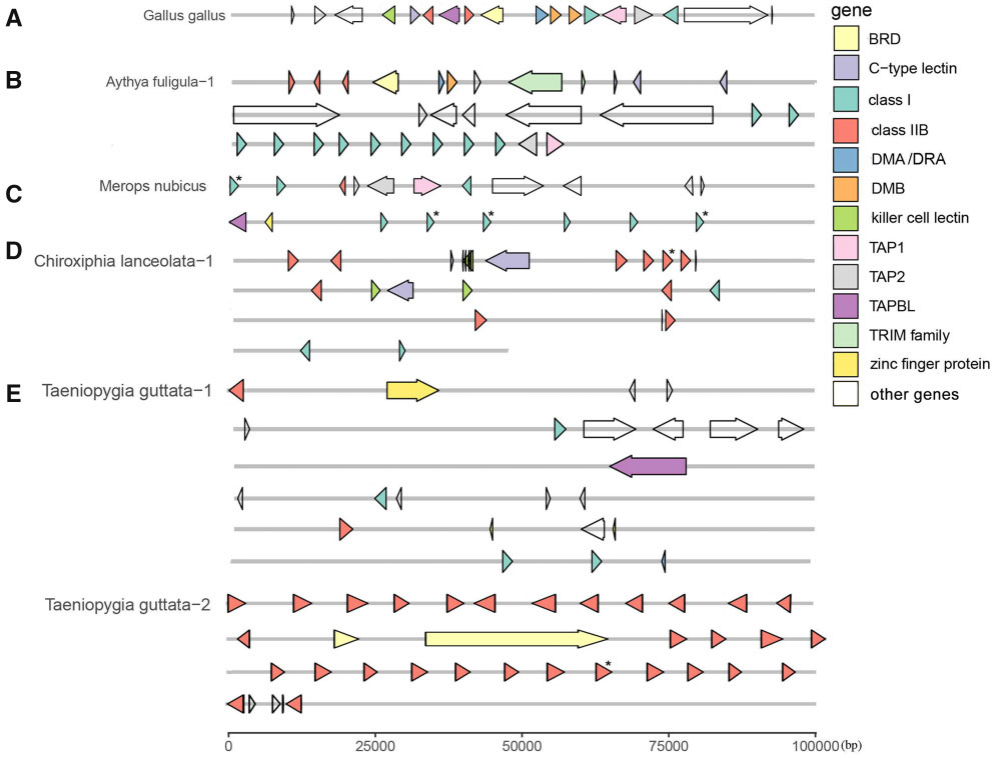
Gene structure in (*A*) *Gallus gallus* (AL023516), in comparison with four other species with numerous (>10) loci: (*B*) contig 1 in *Aythya fuligula*, (*C*) contig 1 in *Merops nubicus*, (*D*) contig 1 in *Chiroxiphia lanceolata*, and (*E*) contigs 1 and 2 in *Taeniopygia guttata*. Arrows indicate the direction of transcription. The genes with stop codons (pseudogenes) are marked with *. Some miscellaneous genes, such as *BTN*, *BG*, *C4*, and *flotillin-1*, are outlined without color and listed as “other genes.” Miscellaneous and unlisted gene names can be found in supplementary table S10, Supplementary Material online. Contig 1 of the barn swallow was too long to show (3.5 million bp), so its genes are listed in supplementary table S10, Supplementary Material online.

We also used MAKER to find other MHC-related genes in species with >10 loci in one contig. The most common genes in Passeriformes were in the zinc finger protein family and serine/threonine-protein kinase PAK 1 (supplementary table S9, Supplementary Material online). In passerines, we also found some genes related to the MHC, such as killer cell lectin-like receptor (*KLRG1*) and TAP binding protein (*TAPBL*). However, in nonpasserines, the other genes we found were not related to the MHC (e.g., ATP Binding Cassette Subfamily B Member, Transporter 1, *TAP1*; Transporter 2, *TAP2*, and DM beta, *DMB*; fig. 6).

## Discussion

In this study, we explored the MHC of birds using a combination of genomes based on TGS and shorter sequences from focused MHC studies (e.g., PCR-based amplicons and BAC libraries). Using all available short sequences from GenBank, we developed a set of consensus MHC sequences at the level of avian Orders, and successfully verified with BLAST searches that they can be used to find MHC sequences in published MHC libraries. Then, we used this set of Order level consensus sequences as queries in BLAST searches to predict the number and location of putative MHC loci in the genomes of a broad range of bird taxa, including species that had not previously been studied at the MHC, but had a published genome. Overall, we found that: 1) TGS-based genomes revealed much higher numbers of MHC loci than NGS-based genomes; 2) the number of MHC loci estimated with TGS-based genomes was up to two times greater than previously reported; and 3) the number of class I and II loci was greater in passerines than nonpasserines. Our results provide the first direct evidence from passerine genomes of these large numbers of loci and their pattern of duplication.

### Improved Methods for Studying the Avian MHC

Most previous studies of the MHC in birds have been based on PCR amplification of specific exons, combined with cloning or NGS (Whittingham et al. 2015; Balasubramaniam et al. 2016). However, these methods are laborious and often fail to separate sequences by locus. To circumvent these problems, we took advantage of the more complete MHC assemblies in TGS-based genomes, which allows for more direct estimates of the number and arrangement of MHC loci. Our results were striking, so first we need to address the accuracy of our methods. In our initial test with published MHC libraries from eight species, our estimates of the number of MHC loci using BLAST searches with Order consensus sequences were exactly the same as the published estimates (supplementary table S1, Supplementary Material online). We were even able to find the same pseudogenes previously identified in these libraries (see supplementary section 5, Supplementary Material online). Then, we made comparisons in 17 species with previously published MHC data (from PCR-based studies of populations). Here, we found a strong positive correlation (*r *=* *0.64, *n *=* *12, *P *=* *0.026) between the estimated number of class II loci in published studies and the number from our Order level BLAST searches. Analyses of class I were limited to six genomes and, in this case, we found no significant correlation.

These results were only possible with TGS-based genomes, because few of the NGS-based genomes had an MHC BLAST match (median = 0 loci), whereas TGS-based genomes typically had several matches (median = 4; 2 loci for class I and II, respectively). As a consequence, there was generally no correlation between estimates of the number of MHC loci derived from NGS and TGS genomes. Furthermore, estimates of the number of loci from our BLAST searches of TGS genomes were more accurate predictors of previous population-based estimates (*r *=* *0.64, see above) than using NGS genomes (*r *=* *0.25, *P *>* *0.5). Overall, our results suggest that using BLAST searches of TGS-based genomes is a more accurate method for obtaining estimates of the number of MHC loci in a species than using NGS-based genomes.

One reason for the greater accuracy of our methods may be that our Order level BLAST searches required all three exons to be within 2 kb of each other. For example, in the Hawaiian crow, a high number of hits were detected when we conducted a BLAST search with only the sequence of class II exon 2, 3, or 4 (60, 17, and 62 hits when exon 2, 3, and 4 were searched separately; data not shown), but fewer hits remained in the final estimate (11 copies) when we only considered hits at all three exons within 2 kb. The rest of the hits were considered pseudogenes. Although our methods appear to be a major improvement for finding MHC loci, we should point out that some of the TGS-based genomes did not reveal any MHC loci when using our requirement that all three exons be present (e.g., class I of Indian peafowl and class II for mallard, common tern and red-crested turaco). Thus, we need further study of some of these discrepancies.

The largest discrepancies in the number of previously reported loci and our estimates occurred in the common yellowthroat (7 vs. 3 in class I and 16 vs. 7 in class II) and zebra finch (1 vs. 2.5 in class I and 11 vs. 24.5 in class II). One of the most likely explanations is that in species with high levels of variation in the number of loci between individuals (e.g., common yellowthroat; Whittingham et al. 2015), the individual whose genome was analyzed may have a higher or lower number of loci than the average number of loci examined in a larger population-based study. Zebra finches also appear to have large variation between individuals in the number of MHC loci in a population (Ekblom et al. 2011).

### Diversity and Evolution of MHC Loci

The number of MHC loci was substantially different between species of birds, especially between passerines and nonpasserines. Overall, passerines had more loci than nonpasserines at both class I (median: 5 vs. 2.3, respectively) and at class II (median: 11 vs. 2, respectively). In nonpasserines, most previous studies reported three or fewer MHC loci for both class I and II (excluding the nonclassical loci found in chicken; table 1; Minias et al. 2018a). In addition to supporting this pattern, our study found some nonpasserines with higher numbers of loci (e.g., tufted duck had 11 class I loci). The highest number of loci occurred in a passerine, the golden-collared manakin, which had 27 class I and 193 class II loci. This is a large increase in the maximum numbers of loci from previous studies (33 class I loci in sedge warbler, *Acrocephalus schoenobaenus* and 16 class II loci in common yellowthroat; reviewed in Minias et al. 2018a). Even in the well-studied zebra finch we found over twice as many class II loci, raising the total from 11 to an average of 24.5 loci (table 1, range = 9–42 in different genomes).

The large number of loci in the three species of manakins we examined is extraordinary and deserves closer scrutiny. It is possible that the large number of loci could be an artifact of lower contiguity in the assembly of this region of the genome. Both golden-collared and wire-tailed manakins, in particular, had large numbers of contigs with MHC exons (12–48) and relatively low contig N50 values (0.3–1.6 Mb), compared with other species (average: 1.9–3.3 for number of loci; 10.6 Mb for contig N50; for more details see supplementary section 7, Supplementary Material online). A larger number of shorter contigs could lead to fewer loci (or exons) per contig, but these values were relatively similar for manakins and other species. Thus, there does not appear to be an obvious bias in the assembly of the genomes of manakins that leads to large numbers of loci. Across all species, there was no correlation between our estimate of the number of MHC loci and genome coverage, scaffold N50 or the number of scaffolds for either class I or II. Nonetheless, the issue of genome assembly and quality could be important in some species and should be evaluated in future studies.

Overall, the incredible variation revealed here raises the question: What kind of selection can lead to such different numbers of MHC loci in passerines? Pathogen richness alone seems like an insufficient explanation. Some superfamilies (especially Sylvioidea, Passeroidea, and Muscicapoidea) with dozens of MHC loci, would seem to require a magnitude higher number of pathogens than basal groups, which does not appear biologically feasible (Bentkowski and Radwan 2019). Another possibility is that there are different intrinsic costs of expressing many MHC variants between passerines and nonpasserines (Bentkowski and Radwan 2019). For example, there could be different tradeoffs between MHC and T cell receptor diversity for these groups (Migalska et al. 2019). Sexual selection may also explain some of the variation in MHC loci. Hamilton and Zuk (1982) suggested that female choice for disease resistant males drives the evolution of exaggerated ornaments and mating displays that signal the ability of males to resist infection by pathogens. This selection for disease resistance can also lead to expansion or contraction of MHC gene families (Bentkowski and Radwan 2020). Manakins have elaborate male mating displays and a lek mating system with extreme sexual selection on males (DuVal and Kempenaers 2008). This could be an important driver of the high numbers of MHC loci we see in manakins; however, we should also point out that some grouse (e.g., prairie-chickens) also have lek mating systems and extreme sexual selection, and yet they possess a low number of MHC loci (Eimes et al. 2010), more similar to chickens and other nonpasserines.

### Flexible Gene Structure in Avian MHC

Several studies of nonpasserines have examined the structure of the MHC. For example, there are 1–3 gene copies of class II in the MHC core region of Galliformes (Ye et al. 2012; Eimes et al. 2013), 1–6 class II loci in quail (Hosomichi et al. 2006), and a variable number of MHC class II αβ dyads in crested ibis (*Nippon nippon*; Chen et al. 2015). In passerines, however, there is no detailed information on the genetic structure of MHC, only a rough mapping of contigs in zebra finch (Balakrishnan et al. 2010; Ekblom et al. 2011). Using the TGS-based genomes, we were able to estimate the number and arrangement of MHC loci (e.g., figs. 4 and 5) which led to some novel observations.

First, the MHC structure of passerines was more complex than other Orders of birds, and classical MHC class I and II genes were often not found on the same contig, nor were they always found with related genes, such as *TAPBL*, *Blec*, and *NK* (fig. 6*D* and *E*). In nonpasserines, however, these related genes were found more often on the same contig with class I and II genes (fig. 6*A*–*C*). Thus, the MHC in passerines appears to be even more complex and dispersed than previously appreciated, and it will remain difficult to study until genomes are assembled to chromosome level.

Second, we found that passerines had the highest level of gene duplication and it was primarily due to duplication of class II loci. This was evident at several levels, including the overall number of multilocus contigs per species and the number of loci per contig. For example, there were four class I loci in one contig (supplementary tables S6 and S7, Supplementary Material online) in the TGS genome of zebra finches, whereas Ekblom et al. (2011) found only one class I locus in a zebra finch BAC library. Only one identical locus was detected when we performed a BLAST search in this BAC (TGAC-102M22), but several unknown nucleotides existed near this locus in the BAC, which might be the location of the other loci. This discrepancy illustrates that TGS genomes can potentially reveal more loci than genomes based on short-reads. Previous mapping of zebra finch chromosomes using FISH suggested that one chromosome had both class I and II loci, and another chromosome had some class II genes (Balakrishnan et al. 2010). Consistent with these earlier results, we found both class I and II loci on a single contig (RRCB01000109.1; supplementary table S7, Supplementary Material online) and several class II loci in a second chromosome (TGAA-157B03 in Balakrishnan et al. 2010). We also found multiple gene duplications in other species, including barn swallow (19 copies of class II in contig 1 and six copies of class II in contig 5), lance-tailed manakin (10 copies of class II in contig 1), and wire-tailed manakin (five copies of class II in contig 13), which provides direct evidence of gene duplication.

Third, we found that both class I and class II genes are sometimes duplicated together (see the shaded blocks in fig. 5A and parallel lines in supplementary fig. S2*A*, Supplementary Material online), although this seems to be relatively uncommon, because it was only detected in the golden-collared manakin. Overall, there appears to be tremendous variation in the arrangement of MHC genes in passerines that has yet to be explored.

Evolution of the number of MHC loci appears to be a complex process in which new genes are produced by duplication followed by selection, drift and mutation, which can convert or delete genes (Nei et al. 1997; Goebel et al. 2017). Previous studies of substitution patterns at the class I and II peptide binding region indicate that there is stronger diversifying (*d*_N_/*d*_S_ > 1) selection at class I of passerines than nonpasserines, but the pattern is reversed at class II (i.e., stronger selection in nonpasserines; Minias et al. 2018b). Comparative analyses of macro-evolutionary patterns also indicate different types of selection have acted on the number of class I (stabilizing) and class II (fluctuating) loci in birds (Minias et al. 2018a). If variation in the number of loci is positively related to patterns of selection, then we would expect higher numbers of class I loci in passerines than nonpasserines, and higher numbers of class II loci in nonpasserines than passerines. However, in our study, we found that there were more loci in passerines than nonpasserines at both class I and II, consistent with the results of Minias et al. (2018a). Thus, the number of loci is likely influenced by a variety of factors including drift and mutation, in addition to selection. Evidence of this can be seen in several evolutionary transitions from high to low numbers of loci, such as the shift from high numbers of class II loci in manakins (a suboscine family) to lower numbers in more derived passerines.

## Conclusion

Overall, the application of long-read sequencing to the study of the avian MHC has confirmed some previous patterns based on more limited types of data, such as the higher number of loci in passerines than nonpasserines. We also discovered much higher numbers of loci in some previously unexplored groups (manakins) and showed that the MHC in birds is much more dispersed in passerines than in nonpasserines, particularly Galliformes. Although long-read sequencing provides some promising advances in resolving the architecture of the avian MHC, there is still a need for more genomes with chromosome level assembly so we can better determine the overall location and spacing of genes, which will allow more rigorous tests of the mechanisms producing MHC diversity (Goebel et al. 2017).

## Supplementary Material

Supplementary data are available at *Genome Biology and Evolution* online.

## Supplementary Material

evaa270_Supplementary_Data

## Data Availability

Sequences used in the study are in the supplementary material along with their GenBank accession numbers (listed in supplementary table S3, Supplementary Material online), and also in Dryad (doi:10.5061/dryad.37pvmcvfj).
