# Supplementary material for: Long-Read Genome Assemblies Reveal Extraordinary Variation in the Number and Structure of MHC Loci in Birds
Source: Genome Biol Evol. 2020 Dec 26;13(2):evaa270. doi: 10.1093/gbe/evaa270 (PMC7875000; doi:10.1093/gbe/evaa270)
Supplement: evaa270_Supplementary_Data [file evaa270_supplementary_data.zip › Suppl-19Nov_HK的副本.docx]

fig S1. The dotplot of (A) *Manacus vitellinus* and (B) *Taeniopygia guttata*. Areas of gene duplication (colored regions in fig S1-A) and gene reversal are marked. The MHC of the zebra finch was found on two multilocus contigs; class I genes were only distributed on multilocus contig 1 and were spaced more than 140kb apart (suggesting no strong linkage), so for this species we performed the self-dotplot on multilocus contigs 2 to 4.

**Section 1. Download of MHC sequences from NCBI.**

**Section 2. Producing a consensus sequence set of MHC class I and II loci.**

**Section 3. Comparison of Order consensus Blast and within-species Blast.**

**Section 4. Consensus sequences for (A) class I and (B) class II loci.**

**Section 5. Pseudogenes found by Blast searches of MHC libraries**

**Section 6. Results of the blastN search in the mallard.**

**Section 7. Details of the number of MHC genes in manakins**

**Section 8. Results of blastN searches in genomes in which we found “zero” MHC loci.**

**Section 9. Arrangement of MHC loci after excluding stop codons.**

**Section 1. Download of MHC sequences from NCBI.**

We downloaded all the avian MHC class I and class II sequences from GenBank (Step 1 in Fig. 1), using “MHC”, “class I” (or “class II”) and “mRNA” as search terms (searched on April 20, 2019). The search was also confined to sequences from 200-2000 bp to exclude very short sequences and long genome scaffolds. We excluded uncertain sequences, which could not be accurately predicted by automatic computational analysis at NCBI, by using “NOT predicted” in the search. We searched for all three exons (2-4) in both class I and class II, excluding pseudogenes with stop codons and known non-classical genes (e.g., Y locus in chickens, *Gallus gallus*), because they may cause errors in alignment and more degenerate sites.

**Section 2. Producing a consensus sequence set of MHC class I and II loci.**

We performed sequential alignments of the sequences at three levels, first within species (Step 2 in Fig 1), then within each Order (or Orders, Step 3 in Fig 1), and finally across all Orders (ie, all species, Step 4 in Fig 1) to produce the final “consensus” sequences at the Order level (Fig. 1). To choose sequences representative of each species (step 2 in Fig 1), we selected sequences from the MUSCLE alignment that had at least 85% nucleotide similarity. If a cluster of similar sequences had more than one sequence, then we randomly chose one of them for clustering at the next (Order level) step (Step 3 in Fig. 1). For the within-species alignment we clustered sequences at a >85% threshold level of nucleotide similarity and for the within-Order alignment we used a >80% threshold of nucleotide similarity (Fig. 1). These two levels of nucleotide similarity were chosen based on the average level of similarity within species and Orders. Higher threshold settings led to many more sequences for the subsequent steps and more degenerate bases in the final consensus sequences (see below).

After all the selected sequences were grouped for each Order (Galliformes, Anseriformes, Passeriformes and all other Orders; Step 3 in Fig 1), we performed a final alignment and estimation of consensus sequences using a 50% majority consensus threshold in Geneious (Step 4 in Fig 1). We chose the 50% threshold after testing six different threshold levels for majority consensus (0 to 95%) using chicken and zebra finch as examples. In brief, the 50% threshold was chosen because it contained all the known alleles when tested in chicken (see the proportion of hits in Table Section 2-A), and in zebra finch the 50% threshold resulted in 7 matches that were missed at the 75% threshold (see Table Section 2-B below). At the end of these steps, we had a consensus sequence set consisting of 8 class I sequences and 12 class II sequences, which were then used as query sequences in BLAST searches of third generation genomes of various species (Section 4-A).

Table Section 2-A. Results of different consensus sequence in *Gallus gallus*

| Threshold (%) | 0 (Majority) | 25 | 50 (Strict) | 75 | 85 | 95 |
| --- | --- | --- | --- | --- | --- | --- |
| No of degenerate bases (exon3) | 3 | 3 | 3 | 22 | 76 | 76 |
| Proportion of degenerate bases (%) | 1.08 | 1.08 | 1.08 | 7.97 | 27.54 | 27.54 |
| Proportion of hits chicken allele sequences (%) | 100 | 100 | 100 | 100 | 96.79 | 96.79 |

Table Section 2-B. Additional Blast hits found using the 50% versus 75% threshold consensus sequences in zebra finch.

| No | query | contig | similarity | length | Location-start | Location-end | e-value | score |
| --- | --- | --- | --- | --- | --- | --- | --- | --- |
| 1 | Passeriformes-3 | CM012106.1 | 67.725 | 189 | 186318 | 186498 | 3.28E-08 | 63.5 |
| 2 | Passeriformes-3 | RRCB01000056.1 | 67.895 | 190 | 565938 | 565755 | 2.69E-09 | 66.2 |
| 3 | Passeriformes-3 | RRCB01000083.1 | 67.895 | 190 | 26592 | 26409 | 2.69E-09 | 66.2 |
| 4 | Passeriformes-3 | RRCB01000109.1 | 65.158 | 221 | 97987 | 98205 | 1.40E-06 | 57.2 |
| 5 | Passeriformes-2 | RRCB01000109.1 | 77.647 | 85 | 617182 | 617098 | 1.07E-11 | 74.3 |
| 6 | Passeriformes-3 | RRCB01000109.1 | 72.596 | 208 | 617267 | 617060 | 1.14E-26 | 124 |
| 7 | Passeriformes-5 | RRCB01000109.1 | 69.681 | 188 | 617267 | 617080 | 1.51E-16 | 92.4 |

**Section 3. Comparison of Order consensus Blast and within-species Blast.**

**Method:**

Except the Order consensus Blast mentioned in MS, we also conducted Blast search with species-specific sequences (named within-species Blast). We call our Blast searches “within-species Blast” and “Order consensus Blast” to indicate that the query sequences came from the same species as the library or genome (although not the same individual). When doing Blast in TGS-based genomes with MHC data, we obtained these amplicon-based estimates of the MHC loci number from our previous study (Minias et al. 2018a) and updated them from NCBI as needed. The species-specific sequences were generated using the PCR-source sequences for each species (results in step 2 of fig 1, supplementary table S4, Supplementary Material online). For two species (Hawaiian crow, *Corvus hawaiiensis*, and New Caledonian crow, *C. moneduloides)*, we used data published in previous MHC studies of Corvus (Eimes et al. 2015); this includes both the estimated number of MHC loci and sequences for Blast. There were more than one thousand sequences of chicken in GenBank, so we randomly chose 60 sequences of class I and class II to search the chicken TGS genome.

The threshold values for matches using “within-species blast” were e-values < 1e-5 and >80% identity over >80% of the length of the query (because MHC sequences and genomes from the same species were used). While for “Order consensus” searches, we reduced the threshold for matches to >60% percent identity over 60% of the alignment to ensure that we would retain likely matches at the Order level where the sequences are less likely to be similar. For some Orders the clustering of various sequences resulted in more than one consensus sequence for the Order (see fig. 1). In these cases, we queried all of the sequences against each genome and used the Blast results with the longest and most similar matches to the query sequences. We also performed Blast searches using both the nucleotide and translated sequences (BlastN and tBlastN, respectively) in within-species blast searches in order to find and exclude pseudogenes.

**Results:**

Among the species with third generation genomes, there were 17 species with data from separate MHC studies of class I or II using amplicon-based technology (Table Section 3-A). Nine of these species had data for both class I and II, so there was a total of 26 data points (counting class I and class II data for both TGS-based genome and published MHC sequences separately; see section 6 of the Supplementary Material online for discussion of data from the mallard).

For within-species Blast, BlastN and tBlastN gave nearly the same estimated number of loci (*r* = 1.0, *n* = 24, *p* < 0.001, Fig Section 3), so we chose tBlastN for further analysis. The number of estimated MHC loci from within-species tBlastN searches was highly correlated with the Order level estimates for both class I (*r* = 0.83, *n* = 12, *p* < 0.001, Fig Section 3) and class II (*r* = 1.0, *n* = 16, *p* < 0.001, Fig Section 3).

The relatedness between estimates of the number of MHC loci by within-species Blast and published numbers were similar. The relatedness were positively correlated in class II (*r* = 0.62, *n* = 12, *p* = 0.032), and not significant in class I (*r* = 0.54, *n* = 6, *p* = 0.27).

Table Section 3-A Predicted number of MHC class I and class II in TGS-based genomes using within species blast and Order consensus blast. Numbers outside the parentheses are the total number of loci, including sequences with stop codons (pseudogenes), while the numbers inside the parentheses are just the number of loci with stop codons. We used dash lines to indicate there is no within species blast in that case.

|  | Class I | | Class II | |
| --- | --- | --- | --- | --- |
| species | within species blast | Order consensus blast | Within species blast | Order consensus blast |
| *Anas platyrhynchos* | 2 | 1 | 0 | 0 |
| *Aythya fuligula* | 13^A^ | 11 | 3^A^ | 3 |
| *Cygnus olor* | 2^A^ | 2 | 2^A^ | 2 |
| *Centrocercus minimus* | 2 | 2 | 3 | 3 |
| *Gallus gallus* | 2 | 2 | 2 | 2 ^B^ |
| *Pavo cristatus* | 0 | 0 | 1 | 1 |
| *Phoenicopterus ruber* | 12 | 8 | 2 | 2 |
| *Balearica regulorum* | -- | | 2 | 2 |
| *Grus nigricollis* | 3^A^ | 3 | 2 | 2 |
| *Aquila chrysaetos* | 3^A^ | 3 | 3 | 3 |
| *Melopsittacus undulatus* | -- | | 1 | 1 |
| *Strigops habroptila* | -- | | 1 | 1 |
| *Corvus hawaiiensis* | -- | | 11^A^ | 11 |
| *Corvus moneduloides* | -- | | 5^A^ | 4 |
| *Geothlypis trichas* | 3 | 3 | 7 | 7 |
| *Hirundo rustica* | 6 | 12 (6) |  | 43 (10) |
| *Taeniopygia guttata* | 4 | 2.5 ^C^ | 28.25 ^C^ | 24.5 (3) |

A) The number of loci was estimated from published studies of the same genus.

1. For chicken, our estimate (2) excludes four non-classical loci with lower similarity.

C) For zebra finch, the number of loci were estimated from the average of several genomes (GCA_000151805.2, GCA_002008985.2, bTaeGut1.pri.cur.20181023.fasta.gz and bTaeGut2.pri.cur.20181019.fasta.gz).

Fig Section 3. The results of three methods (within-species blast, within-species tblastN and Order blasts) in TGS-genome with published MHC data. (A) the same results obtained by three methods in class I of A*quila chrysaetos,* *Centrocercus minimus, Cygnus olor, Gallus gallus, Geothlypis trichas*, *Grus nigricollis, Sterna hirundo* and *Taeniopygia guttata,* and class II of *Aquila chrysaetos*, *Aythya fuligula, Balearica regulorum, Centrocercus minimus, Cygnus olor, Geothlypis trichas, Grus nigricollis, Merops nubicus, Pavo cristatus* and *Phoenicopterus ruber*; (B) class I of *Hirundo rustica*; (C) class II of *Gallus gallus;* (D) class II of *Corvus hawaiiensis*; (E) class II of *Taeniopygia guttata*; (F) class II of *Aythya fuligula*; (G) class II of *Corvus moneduloides*; and (H) class I of *Phoenicopterus ruber*.

**Section 4. Consensus sequences at the Order level for (A) class I and (B) class II loci. Exons 2, 3 and 4 are marked in yellow, green and purple.**

Sequences were produced from 18 species in 13 families (two sequences for Galliformes, one for Anseriformes, one for other non-passerine Orders and four for Passeriformes). For class II we generated 12 consensus sequences (157-747 bp) from 41 species in 21 families (three for Galloanserae, six for Passeriformes and three for other avian Orders). Galliformes and Anseriformes were combined into Galloanserae because the sequences had similarity >80%. Fasta files with the sequences can be found on Dryad (doi:10.5061/dryad.37pvmcvfj) (deposited upon acceptance). Dots (.) indicate nucleotides identical to the majority consensus sequence (top row) and dashes indicate gaps in the alignment. Blanks indicate no bases in that region. Note that there are multiple versions of the Order level sequence for some Orders (eg, two for Galliformes at class I). These were caused by higher levels of sequence variation in these groups. Each Order has one primary sequence with all three exons (2, 3, 4), but the additional sequences may be missing exons (in whole or part) because of incomplete data.

A


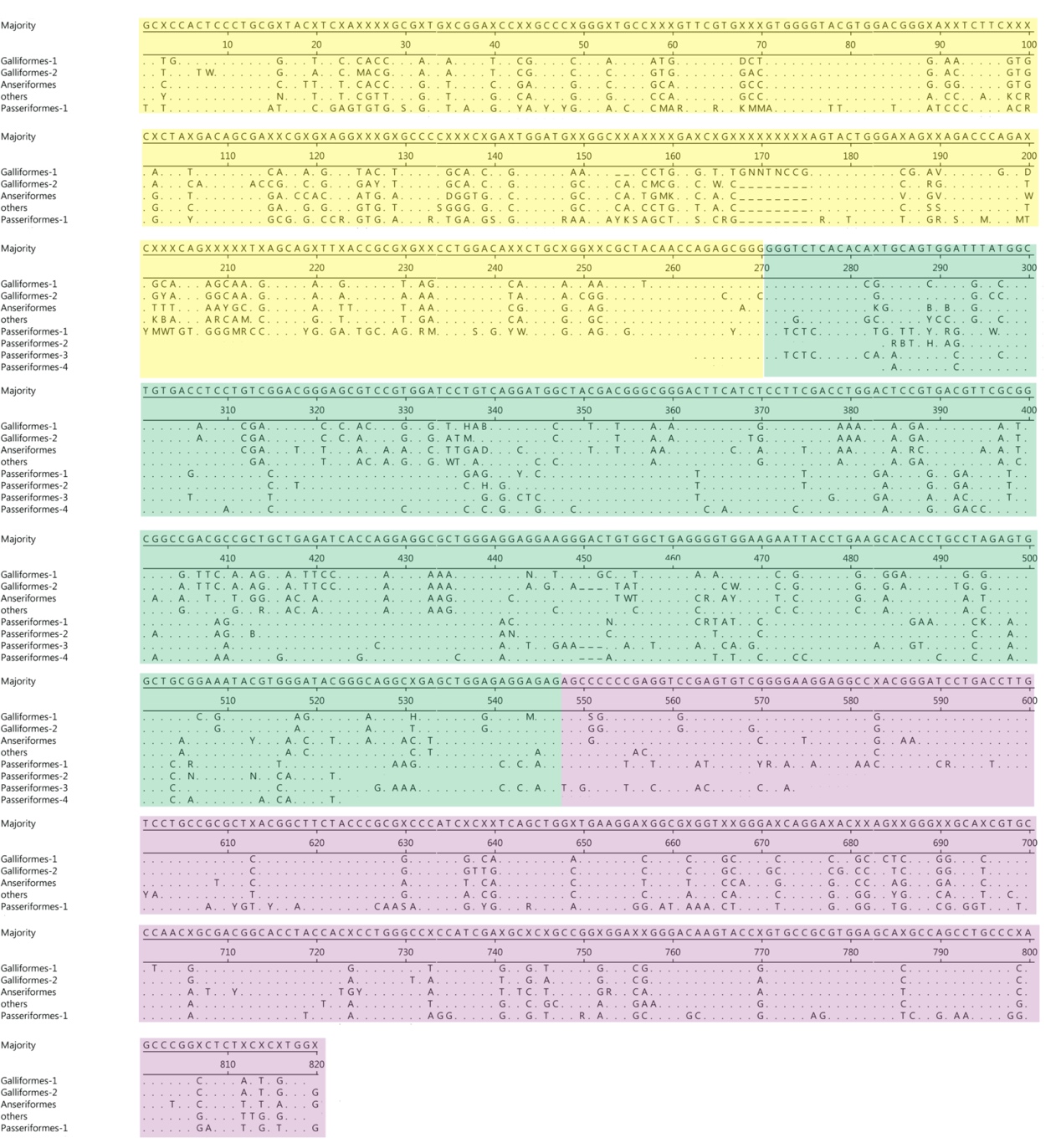


B


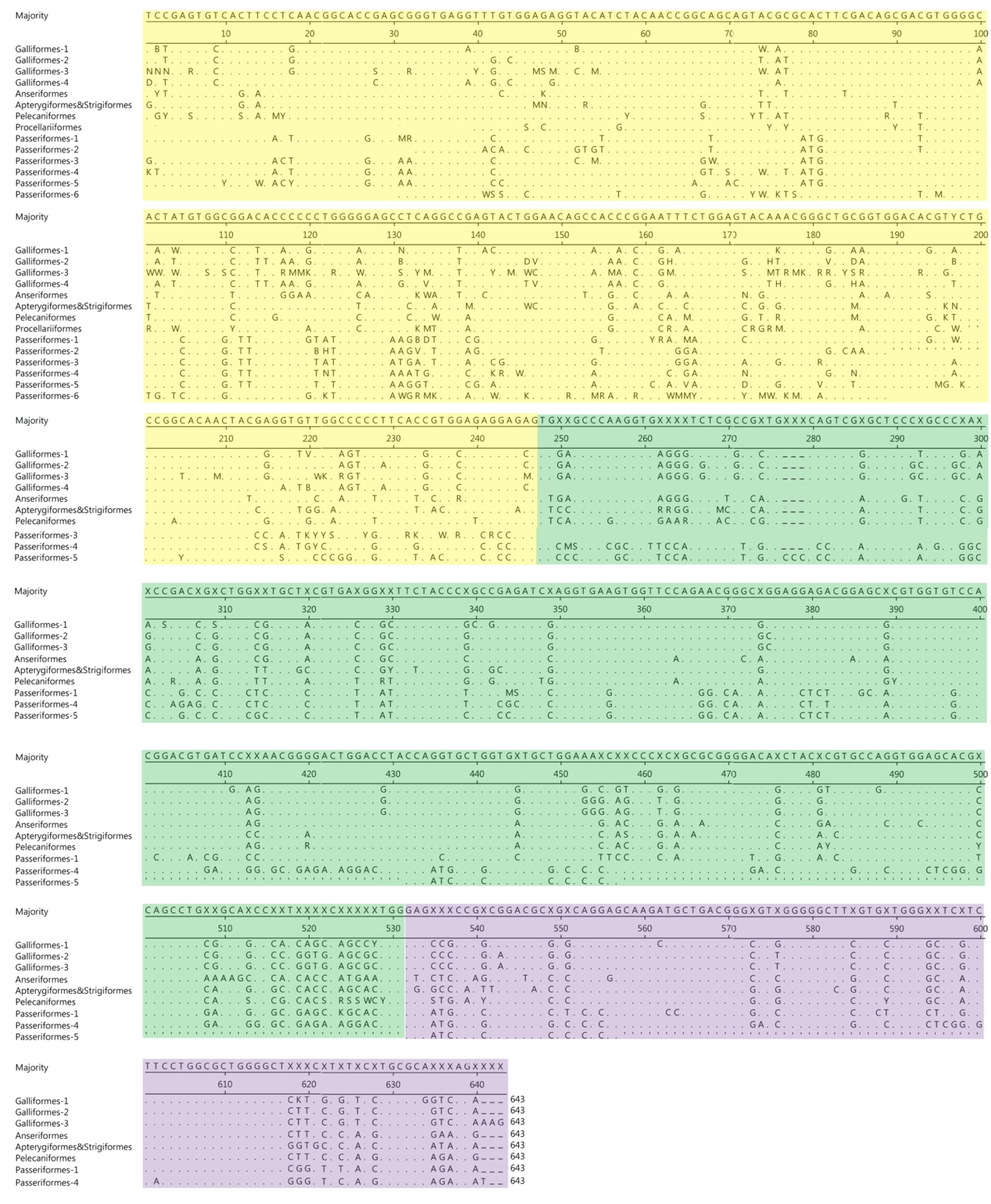


**Section 5. Pseudogenes found by Blast searches of MHC libraries**

We found three cases of pseudogenes in our Blast searches of MHC libraries. These included an exon of the Japanese quail (AB078884, Coja-F) and Oriental stork (LC180358, UBA1 and UBA2), and another in the mallard (a premature stop codon; AY885227, UCA). Thus, our BlastN searches using the Order-consensus sequences can detect pseudogenes with missing exons and low similarity, as shown in the quail and stork examples. Nonetheless, there were still some pseudogenes remaining that were caused by premature stop codons with little change in length (UCA in mallard). Excluding these additional loci with stop codons had little qualitative effect on our results.

**Section 6. Results of the BlastN search in the mallard.**

The estimated number of MHC loci in mallards in Table 1 was zero for class II, because we required all three exons (2, 3, and 4) to be found in a match. Below we summarize the partial matches of our consensus sequences with all 4 *Anas platyrhynchos* genomes in NCBI (Table Section 6-A). The first column corresponds to the results shown in Table 1 with a TGS (PacBio) genome. Note that two genomes based on Illumina sequencing (Illumina, Hiseq) show a >2x difference in the number of class II loci (6 vs 12-13). These differences might be related to differences in coverage of the SGS-based genomes or differences in the number of duplicated loci between individuals.

Table Section 6-A. The estimated number of MHC loci in mallards using blastN searches and our consensus sequences in four different genomes. Results are shown for searches using all exons (2-4) or just some (exon 2 or 3).

| Assembly ID | GCA_003850225.1 | GCA_002743455.1 | [GCA_000355885.1](https://www.ncbi.nlm.nih.gov/assembly/GCA_000355885.1) | GCA_002224895.1 |
| --- | --- | --- | --- | --- |
| Sequencing (year) | PacBio (2018) | Illumina (2017) | Solexa (2013) | Hiseq (2017) |
| Coverage | 50 | 150 | 60 | 208 |
| class I own-blast-Exon3 | 2 | 9 | 2 | 13 |
| Class I own-tblastN-Exon3 | 2 | 9 | 2 | 13 |
| Class I Order consensus-blast (exon3) | 2 | 9 | 2 | 13 |
| Class I Order consensus-blast (exon2-4) | 1 | 4 | 1 | 10 |
| class II own-blast-Exon2 | 0 | 6 | 4 | 13 |
| Class II own-tblastN-Exon2 | 0 | 6 | 4 | 13 |
| Class II Order consensus-blast (exon2) | 0 | 6 | 4 | 12 |
| Class II Order consensus-blast (exon2-4) | 0 (only exon3 got 7 hits) | 0 | 1 | 5 |

**Section 7. Details of the number of MHC genes in manakins**

We examined the MHC genes of manakins in more detail because of their high number of loci. The table below has a summary of our results. The first pattern to note is that manakins have a larger number of contigs than other species at both class I (Kruskal-Wallis test *p* = 0.016) and II (*p* = 0.03). This pattern also tends to hold if you look at just the number of contigs with a single locus or 2+ loci (multilocus), although it is not always significant. It is possible that the density of exons per contig differs between species, so next we examined the number of exons on each contig, in particular, exons 2 and 3, which encode the peptide binding regions in class I and class II (exon 2 only). Although there are more contigs in manakins, there are about the same number of exons 2 and 3 on each contig (1.3-6.5 exons per contig in manakins vs 2.5-3 in other species). The distribution of exons per contig for each species is shown in the graphs below. These graphs illustrate that manakins are not too extreme in terms of the number of exons (2 and 3) per contig.

One possible explanation for the large number of contigs in manakins is that the MHC region is not as well assembled as in some other long-read based genomes, so there are more contigs and fewer loci per contig. The lower contiguity of the golden-collared manakin (*M. vitellinus*) assembly is evident in the contig N50, which is the lowest of any passerine in our sample (0.29 Mb; average for all species = 10.6 Mb). *P. filicauda* also has a relatively low contig N50 (1.6 Mb), but *C. lanecolata* is above average (18.5 Mb).

Table Section 7-A. Comparison of the number of contigs and exons (2 & 3) in manakins and other species (original data in Table S7). Note that there was only one case where an exon (2 or 3) was missing on a contig that contained other exons (ie, class II exon 2 was missing from *Corvus moneduloides* on scaffold 118, but it contained exon 3).

|  | number of unique contigs (class I, II) | N of contigs with a single locus (class I, II) | N of contigs with 2+ loci (multilocus cluster) (class I, II) | Number of exons (2 & 3) per contig in class I, II (includes single locus contigs) |
| --- | --- | --- | --- | --- |
| Other species (average, n=29) | 1.9, 3.2 | 0.9, 1.9 | 1.0, 1.3 | 3, 2.5 |
| All manakins (ave, n=3) | 11.7, 63.7 | 9.3, 49 | 2.3, 14.7 | 1.8, 1.7 |
| Lance-tailed manakin *Chiroxiphia lanceolata* | 2, 2 | 0, 0 | 2, 2 | 2.5, 6.5 |
| Wire-tailed manakin *(P.filicauda)* | 12, 41 | 10, 25 | 2, 16 | 1.25, 1.7 |
| Golden-collared manakin *Manacus vitellinus* | 21, 148 | 18, 122 | 3, 26 | 1.29, 1.3 |


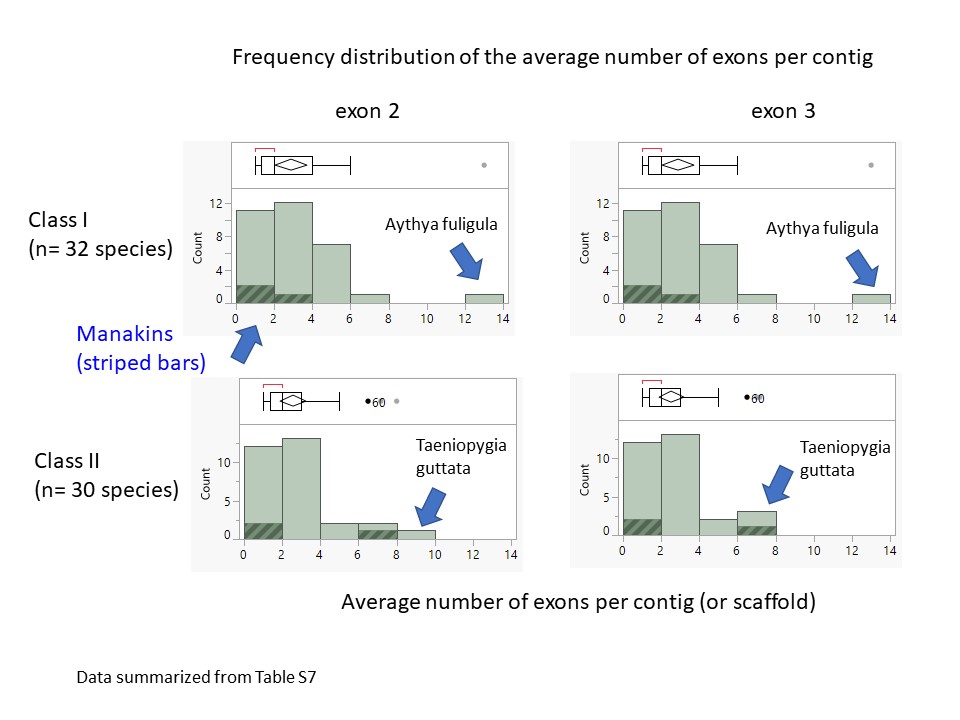


**Section 8. Results of BlastN searches in genomes in which we found “zero” MHC loci.**

We found zero MHC loci in class I of *Pavo cristatus,* as well as class II for A*nas platyrhynchos,* *Sterna hirundo,* *Tauraco erythrolophus* and *Streptopelia turtur.* However, our criterion for counting MHC loci required finding matches with all three exons (2, 3 and 4). As indicated below, there were some partial matches in each species.

Table Section 8-A. Summary of Blast results in species with “zero” MHC matches in searches of genomes.

| species | query | hits number | contigs | similarity | length | start | end | e-value |
| --- | --- | --- | --- | --- | --- | --- | --- | --- |
| *Anas platyrhynchos* | ClassII-exon2 | 0 |  |  |  |  |  |  |
|  | ClassII-exon3 | 6 | NW_020866000.1 | 74.038 | 104 | 10083 | 9980 | 4.74e-09 |
|  |  |  | NW_020865945.1 | 74.51 | 102 | 9244 | 9146 | 2.02e-07 |
|  |  |  | NW_020865945.1 | 73.786 | 103 | 13321 | 13223 | 8.58e-06 |
|  |  |  | NC_040062.1 | 74.51 | 102 | 328641 | 328739 | 2.02e-07 |
|  |  |  | NC_040062.1 | 74.51 | 102 | 342682 | 342584 | 2.02e-07 |
|  |  |  | NC_040062.1 | 74.51 | 102 | 449955 | 449857 | 2.02e-07 |
|  | ClassII-exon4 | 0 |  |  |  |  |  |  |
| *Sterna hirundo* | ClassII-exon2 | 0 |  |  |  |  |  |  |
|  | ClassII-exon3 | 2 | scaffold_100\|arrow | 72.581 | 124 | 25959 | 26080 | 3.65e-09 |
|  |  |  | scaffold_100\|arrow | 72.951 | 122 | 20807 | 20927 | 1.27e-08 |
|  | ClassII-exon4 | 0 |  |  |  |  |  |  |
| *Pavo cristatus* | Class I-exon2 | 0 |  |  |  |  |  |  |
|  | Class I-exon3 | 0 |  |  |  |  |  |  |
|  | Class I-exon4 | 4 | QZWQ01019631.1 | 69.519 | 248 | 3592 | 3408 | 8.35e-10 |
|  |  |  | QZWQ01003722.1 | 69.519 | 248 | 3695 | 3879 | 8.35e-10 |
|  |  |  | QZWQ01003722.1 | 69.519 | 248 | 6188 | 6372 | 8.35e-10 |
|  |  |  | QZWQ01003722.1 | 69.519 | 248 | 9600 | 9784 | 8.35e-10 |
| *Tauraco erythrolophus* | ClassII-exon2 | 0 |  |  |  |  |  |  |
|  | ClassII-exon3 | 1 | scaffold_47_arrow_ctg1 | 67.513 | 257 | 37463 | 37267 | 1.58e-07 |
|  | ClassII-exon4 | 0 |  |  |  |  |  |  |
| *Streptopelia turtur* | Class I-exon2 | 0 |  |  |  |  |  |  |
|  | Class I-exon3 | 0 |  |  |  |  |  |  |
|  | Class I-exon4 | 3 | scaffold_11 | 73.016 | 126 | 2392526 | 2392650 | 1.17E-08 |
|  |  |  | scaffold_11 | 73.016 | 126 | 2396831 | 2396955 | 1.17E-08 |
|  |  |  | scaffold_11 | 73.81 | 126 | 2400798 | 2400922 | 1.17E-08 |
|  | ClassII-exon2 | 0 |  |  |  |  |  |  |
|  | ClassII-exon3 | 3 | scaffold_11 | 71.901 | 121 | 2392614 | 2392732 | 6.30E-06 |
|  | ClassII-exon4 | 0 | scaffold_11 | 71.901 | 121 | 2396919 | 2397037 | 6.30E-06 |
|  |  |  | scaffold_11 | 71.901 | 121 | 2404864 | 2404982 | 6.30E-06 |

**Section 9. Arrangement of MHC loci after excluding stop codons.**

We also examined the arrangement of MHC loci in 102 contigs with at least two loci and no stop codons. These multi-locus contigs had an average of 3.8 loci per contig (range: 2 – 30; median = 3 loci) after excluding loci with stop codons. Most species had one (n=12) or two (n=9) multilocus contigs in their genomes, but the manakins contained many more with 18 in wire-tailed manakin and 26 in golden-collared manakin. Only 34 of the 102 contigs had both class I and class II loci, and these were found equally often in passerines (median =1 per species) and non-passerines (median =0 per species; Χ 2 = 1.1, *P*=0.28). Overall, there were more multilocus contigs per species in passerines (median =2.5) than non-passerines (median =1; Χ 2 = 8.3, *P*=0.004), and these were most often contigs with just class II loci, primarily because of the large number in wire-tailed (n=14 class II contigs) and golden-crowned (n=20 class II cointigs) manakins.
