# Supplementary figures and images for: Long-Read Genome Assemblies Reveal Extraordinary Variation in the Number and Structure of MHC Loci in Birds
Source: Genome Biol Evol. 2020 Dec 26;13(2):evaa270. doi: 10.1093/gbe/evaa270 (PMC7875000; doi:10.1093/gbe/evaa270)

**A** *Manacus vitellinus*

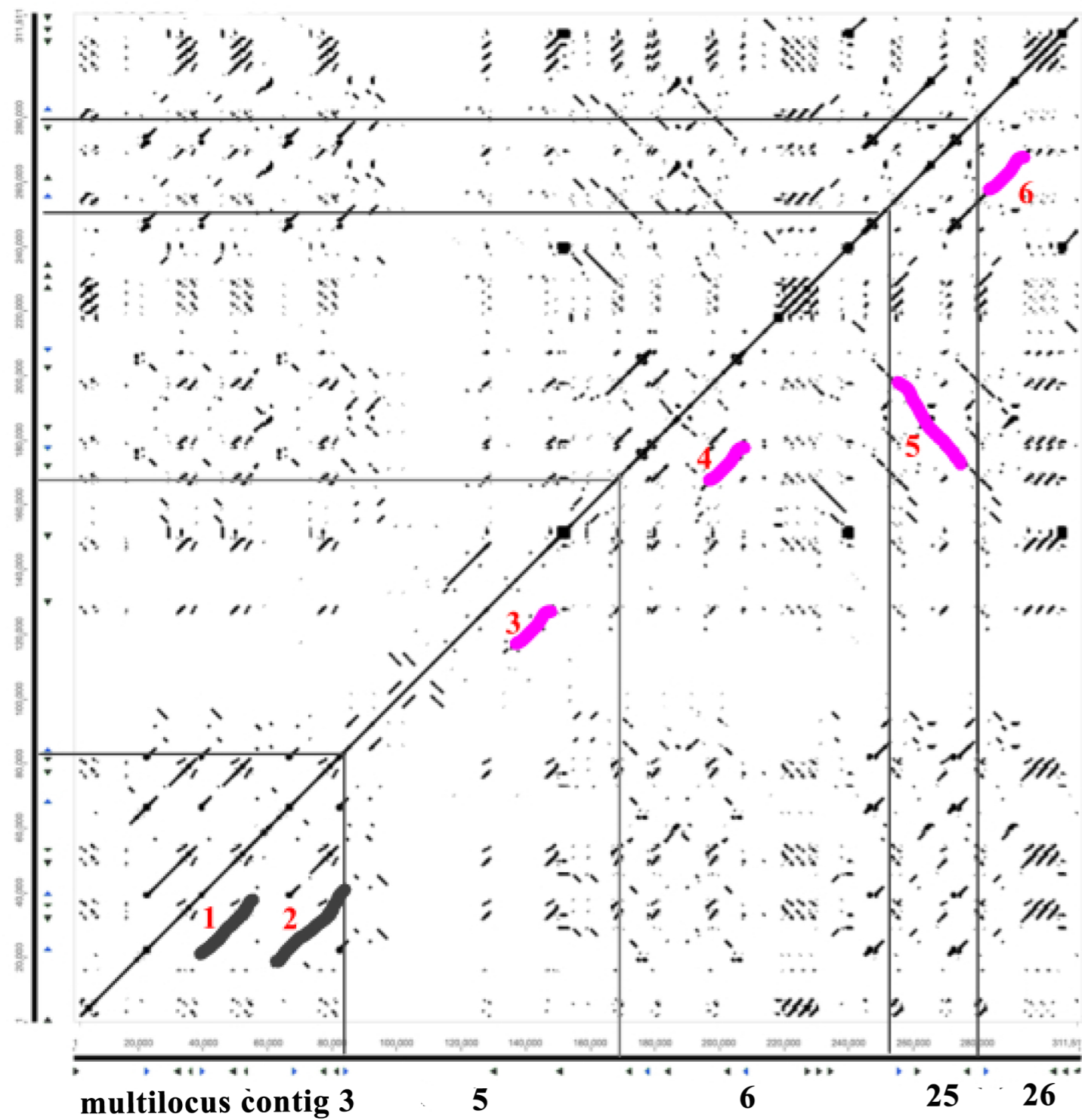

**B** *Taeniopygia guttata*

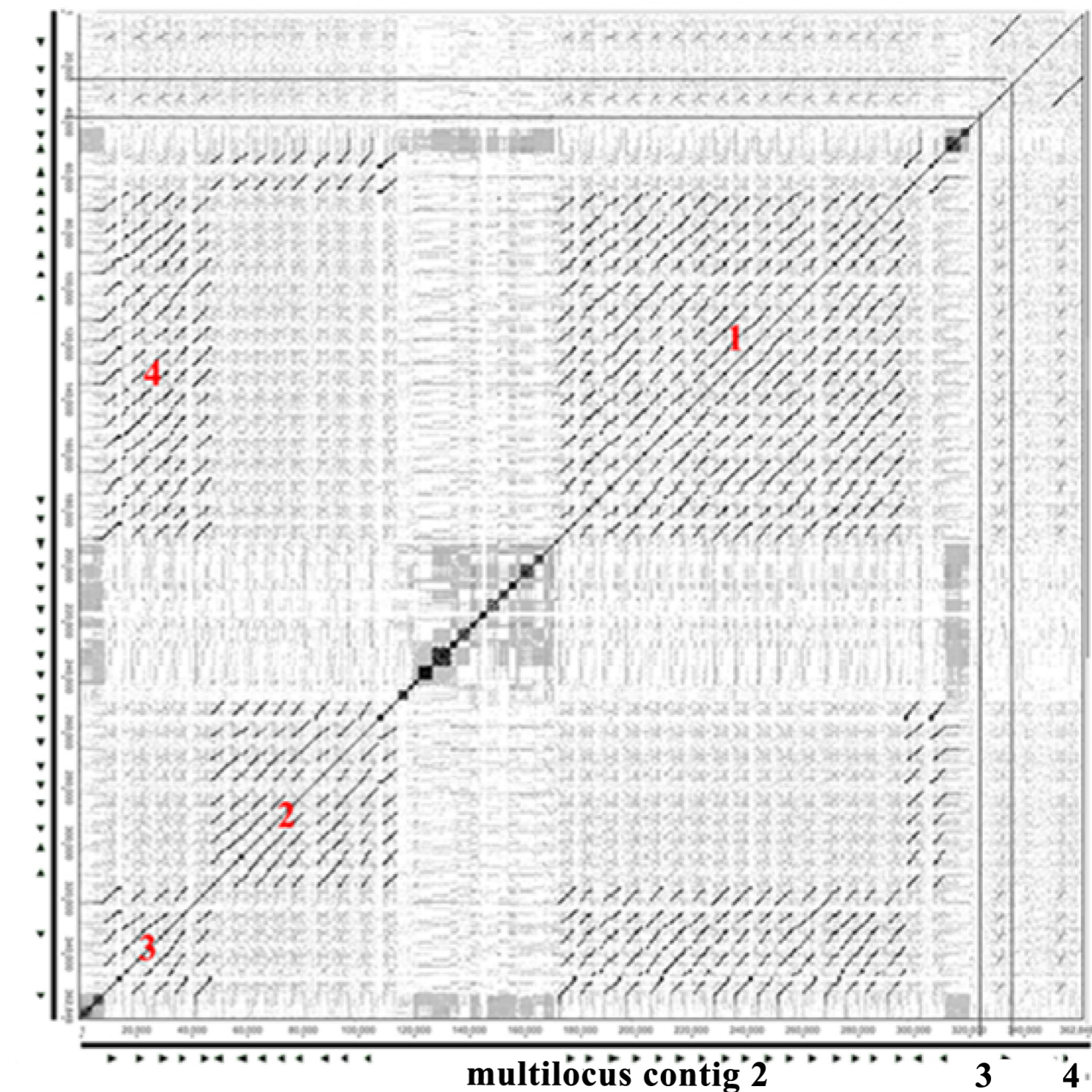

Supplement: evaa270_Supplementary_Data [file evaa270_supplementary_data.zip › Suppl-FigS1(previous S2).pdf]
